# Supplementary material for: Genome-wide identification, characterization and gene expression of BES1 transcription factor family in grapevine (Vitis vinifera L.)
Source: Sci Rep. 2023 Jan 5;13:240. doi: 10.1038/s41598-022-24407-y (PMC9816167; doi:10.1038/s41598-022-24407-y)
Supplement: Supplementary file 3 — Supplementary Information. [file 41598_2022_24407_MOESM3_ESM.zip › Vvi_Atr/Vitis_vinifera.PN40024.v4.dna_sm.toplevel.fa.vs.Amborella_trichopoda.AMTR1.0.dna_sm.toplevel.fa.html/Atr-AmTr_v1.0_scaffold00194.html]

|  |  |  |  |  |  |  |  |  |  |  |  |  |  |
| --- | --- | --- | --- | --- | --- | --- | --- | --- | --- | --- | --- | --- | --- |
| Duplication depth | Reference chromosome | Collinear blocks | | | | | | | | | | | |
| 0 | Atr-ERN18968 |  |  |  |  |  |  |  |  |  |  |  |  |
| 0 | Atr-ERN18969 |  |  |  |  |  |  |  |  |  |  |  |  |
| 0 | Atr-ERN18970 |  |  |  |  |  |  |  |  |  |  |  |  |
| 0 | Atr-ERN18971 |  |  |  |  |  |  |  |  |  |  |  |  |
| 0 | Atr-ERN18972 |  |  |  |  |  |  |  |  |  |  |  |  |
| 0 | Atr-ERN18973 |  |  |  |  |  |  |  |  |  |  |  |  |
| 0 | Atr-ERN18974 |  |  |  |  |  |  |  |  |  |  |  |  |
| 0 | Atr-ERN18975 |  |  |  |  |  |  |  |  |  |  |  |  |
| 0 | Atr-ERN18976 |  |  |  |  |  |  |  |  |  |  |  |  |
| 0 | Atr-ERN18977 |  |  |  |  |  |  |  |  |  |  |  |  |
| 0 | Atr-ERN18978 |  |  |  |  |  |  |  |  |  |  |  |  |
| 0 | Atr-ERN18979 |  |  |  |  |  |  |  |  |  |  |  |  |
| 0 | Atr-ERN18980 |  |  |  |  |  |  |  |  |  |  |  |  |
| 0 | Atr-ERN18981 |  |  |  |  |  |  |  |  |  |  |  |  |
| 0 | Atr-ERN18982 |  |  |  |  |  |  |  |  |  |  |  |  |
| 0 | Atr-ERN18983 |  |  |  |  |  |  |  |  |  |  |  |  |
| 0 | Atr-ERN18984 |  |  |  |  |  |  |  |  |  |  |  |  |
| 0 | Atr-ERN18985 |  |  |  |  |  |  |  |  |  |  |  |  |
| 0 | Atr-ERN18986 |  |  |  |  |  |  |  |  |  |  |  |  |
| 0 | Atr-ERN18987 |  |  |  |  |  |  |  |  |  |  |  |  |
| 0 | Atr-ERN18988 |  |  |  |  |  |  |  |  |  |  |  |  |
| 0 | Atr-ERN18989 |  |  |  |  |  |  |  |  |  |  |  |  |
| 0 | Atr-ERN18990 |  |  |  |  |  |  |  |  |  |  |  |  |
| 0 | Atr-ERN18991 |  |  |  |  |  |  |  |  |  |  |  |  |
